# A Review of the Use of Complementary and Alternative Medicines by Children with Inflammatory Bowel Disease

**DOI:** 10.3389/fped.2013.00009

**Published:** 2013-05-01

**Authors:** Andrew S. Day

**Affiliations:** ^1^Department of Paediatrics, University of Otago ChristchurchChristchurch, New Zealand

**Keywords:** Crohn disease, ulcerative colitis, children and adolescents, complementary therapies, communication

## Abstract

The Inflammatory Bowel Diseases (IBDs) are diagnosed more commonly in children and adolescents. Following diagnosis, the key objectives are to achieve and then maintain remission. Although some therapies are able to effectively modify and modulate inflammatory events, none of the available interventions cure these conditions. Consequently, children and their parents face uncertainty and may look to alternative management options as ways to help their child, which may include various complementary and alternative medicines (CAMs). A number of studies have shown that many children with IBD receive or are given CAM agents. This article reviews the rates and patterns of CAM use in children with IBD, and emphasizes the increasing importance of these aspects of the management of children with IBD.

## Introduction

The term complementary and alternative medicine (CAM) can be defined as “a group of diverse medical and healthcare systems, practices, or products that are not generally considered part of conventional medicine” (1). CAMs encompass a wide range of therapies, including nutritional supplements, meditation, and herbal remedies (2, 3). Several of these agents, such as probiotics, may have specific beneficial effects upon the gastrointestinal (GI) tract, including modulation of host-pathogen interactions and anti-inflammatory benefits. Other CAM interventions may improve the individual’s general well-being or enhance the person’s abilities to cope with stressful events.

Such potential benefits may be especially relevant for an individual suffering a chronic illness. Rates of use of CAM agents tend to be higher in individuals with chronic, debilitating, or life threatening illnesses. In one study, for instance, three times as many children with Inflammatory Bowel Disease (IBD) or cerebral palsy were using CAM than healthy children (4). The IBDs are chronic incurable conditions affecting the GI tract, leading to numerous bowel symptoms as well as systemic consequences (5). This article will review available data of the patterns of CAM use in children with IBD.

## Crohns and Colitis

### The inflammatory bowel diseases

Crohn’s disease (CD) and ulcerative colitis (UC) are the two main types of IBD (5, 6). Although these two conditions can be differentiated on the basis of disease location, inflammatory patterns, and specific histological findings, these diseases also have many common features. In particular, they both feature chronic inflammation of the GI tract, lead to an array of intestinal symptoms (such as diarrhea and pain), and both are incurable.

Although the precise causes of IBD are yet fully understood, it is commonly accepted that IBD occurs as a result of interactions between the intestinal mucosa and the intestinal microflora or their byproducts, leading to dysregulated inflammation in a genetically susceptible host. In recent years rates of IBD have increased in many countries, including in areas where IBD was previously seen rarely (7, 8).

Both CD and UC can begin at any age, with around a quarter of individuals diagnosed in childhood (9). Recent data show tenfold increases in UC and CD over a decade in one Australian tertiary center (10, 11).

Published series consistently show that childhood-onset CD and UC differ substantially from the disease presenting in adults (12, 13). Most children diagnosed with UC have pan-colonic involvement at diagnosis with isolated distal changes being uncommon. Children diagnosed with CD tend to have much more extensive disease than adults: pan-enteric disease, especially with upper GI tract involvement, occurs commonly (12).

### Consequences of IBD in children

Crohn’s disease and UC commonly impact adversely upon growth, pubertal development, and daily activities in children. Almost all children with CD, and up to two-thirds of those with UC, have lost weight or grown poorly prior to diagnosis (14). Many children will have impaired linear growth and some will have delayed pubertal development at diagnosis. Furthermore, a large number of children will have ongoing problems with growth, and pubertal development following diagnosis (15).

Growth disruption is consequent partly to impaired dietary intake, but especially to uncontrolled inflammation. The pro-inflammatory cytokine, interleukin (IL)-6 impairs synthesis and activity of insulin-like growth factor (IGF)-1, a key signaling component of the growth hormone pathway (16). In addition, IL-6 and tumor-necrosis-factor (TNF)-α also have direct adverse effects upon growth plate function.

In addition, CD and UC commonly adversely affect the day to day functioning of children or adolescents with these conditions (17). Children may have difficulty playing sports or have interrupted school attendance. Further, depression and anxiety occur at higher rates. These aspects contribute to impaired health related quality of life (QOL) (18).

### Management of pediatric IBD

Whilst the central goals of the management of IBD in children and adolescents are to improve symptoms and resolve inflammation, further objectives include ensuring that children have normal growth and development, and that they are able to undertake regular day to day activities (5). Current standard medical therapies can be divided into those that are used to induce remission and others used to maintain remission. Examples of the former include exclusive enteral nutrition, corticosteroids, and biological therapies (e.g., infliximab). Interventions utilized for maintenance of remission include aminosalicylates, immunosuppressive drugs (e.g., azathioprine), and biological therapies (5). However, none of the available therapies for pediatric CD are curative. In addition, almost all therapies have potential side-effects. Hence, the benefits of any specific therapy need to be weighed against the potential adverse effects of this intervention within the individual patient context.

Almost all children and adolescents will commence a therapy following diagnosis and will have ongoing therapies throughout childhood. Many children will take a number of medicines one or more times each and every day. Despite these interventions, these children remain at risk of relapse of disease, which may be triggered by stressful events or following an inter-current infection.

Because of the persistent and pervasive features of CD and UC in childhood, and in the context of no curable intervention, the parents of many children consider other options, including CAM. The use of CAM therapies, the range of available agents and contributory factors have been evaluated in a number of studies over the last decade.

## CAM Therapies Considered for IBD

A range of CAM therapies have been considered in the context of IBD. Some of these may have particular relevance for individuals with IBD (3). Some CAM therapies have direct anti-inflammatory effects that could enhance disease control in conjunction with standard treatments. These include nutritional interventions, agents that may enhance host immune responses, and interventions that may modulate psychological or emotional responses to the disease. Herbal therapies appear to be one of the most commonly used CAM, but substantial variations occur between cultures and regions of the world.

Langmead and Rampton (3) reviewed the data supporting the benefits of CAM therapies used commonly in the context of IBD. These authors concluded that there is little controlled data supporting agents that have been commonly suggested for IBD. Note was made, however, of promising data arising from studies examining acupuncture in IBD. Hilsden et al. (19) made similar conclusions relating to the evidence supporting CAM in IBD and referred to specific resources, including internet databases of available CAM agents.

## Use of CAM in Adults with IBD

The use of CAM in adults with IBD was reviewed recently, with focus upon reports that included 100 or more adult subjects with IBD (19). Current use of CAM across these studies ranged from 11 to 34%, with up to 60% having either past or current use. The authors identified a range of factors for the use of CAM: these were dependent upon the situation and methodology used to collect data. Overall, however, common factors include disease severity and duration, requirements for standard medications, QOL, and history of surgical intervention and requirement for hospitalization.

One earlier individual study has illustrated variations in the rates and patterns of CAM usage in adults with IBD between countries (20). This study was conducted late last century and used a self-administered questionnaire to assess usage in 218 adults with IBD from two North American centers and two centers in Europe. Rates of CAM use varied between 31 and 68% of subjects, with substantially higher rates seen in North American patients. The respondents in this series most commonly used exercise, prayer, counseling, massage, chiropractic, and relaxation therapies. Respondents reported that the factors supporting their use of CAM included dissatisfaction with standard therapies, unfavorable attitude to healthcare facilities, and sense of hopelessness about their medical condition.

## Use of CAM in Children and Adolescents with IBD

A number of studies have examined the use of CAM by children and adolescents with IBD. Although patterns have been observed, there are also some specific regional and/or cultural variations. Further, there are some particular differences from the patterns of CAM usage seen in adults with IBD.

In general, the decision to administer CAM therapies to children and adolescents will likely be made by a parent or caregiver, rather than the child themselves. Consequently, the decision making process may reflect parental attitudes along with perceived requirements and responses.

We examined CAM usage in Australian children attending general gastroenterology clinics (21, 22) and attending specific IBD clinics (23) using a specific questionnaire. In an initial study of 92 children attending a range of gastroenterology clinics, more than one third of the children were being given CAM (21). Just 10 of this group had been diagnosed with IBD: three of these children were taking CAM. Overall, 90% of the parents of the 92 children reported that they would consider a CAM agent for their child if recommended.

A subsequent report a decade later involved 98 children attending general gastroenterology clinics in the same hospital (22). The children in this cohort were being managed for a variety of GI conditions, but none were diagnosed with IBD. This report demonstrated increased recent or current usage rates (69%) compared to the previous estimates, with wide awareness and acceptance by parents. Nutritional supplements and probiotics were the most commonly used CAM, followed by herbal remedies, massage, fish oil, and relaxation techniques.

In addition, a further questionnaire-based assessment solely of children diagnosed with IBD was conducted in the same hospital (23). This report involved the responses from 46 families following receipt of a mailed questionnaire. Almost three quarters of the children were reported by their parents to be receiving CAM, which was greater than the rate of the children without IBD from the same location. On average these children were receiving 2.4 CAM agents, with four children having five or more therapies. Fish oil and probiotics were the most common agents utilized in these children. Other common interventions included herbal remedies, homeopathy, and supplementary vitamins. Relaxation techniques, massage, and chiropractic interventions were uncommonly utilized. Interestingly only 12% of the CAM-users reported the agents to be very effective – however 50% of the users also noted partial benefits. In this cohort, there were no associations between age, gender, disease type or duration of disease, and CAM usage.

In contrast to the high rates seen in this Australian group, much lower rates were seen in a Canadian cohort (24). Twenty-two percent of this group reported having ever used CAM, whilst just 6.7% reported current use. Other European and North American reports have defined rates between these extremes.

Heuschkel et al. (25) surveyed 208 children seen in two North American centers and one center in the United Kingdom. CAM usage was reported in 41%, with megavitamins, diet supplements, and herbal remedies being most commonly administered. Higher rates of CAM usage were seen in children whose parents also use CAM and those who had more side-effects secondary to standard drugs. Interestingly, 59% of non-users reported an interest in learning more about CAM.

A similar rate of CAM usage was determined in a large cohort of 334 children recruited from one city in the USA (26). Although megavitamins were used commonly, nutritional supplements, probiotics, and fish oil were also frequently reported. The factors associated with use of CAM were explored in more detail in this study. Univariate analysis suggested that CD, access to the internet, poor QOL, and more frequent school absences were factors favoring the use of CAM. Further regression analysis ascertained that poor QOL, use of the internet for research about IBD, and a need for calorie supplementation were specific factors linked with CAM use. In addition, those individuals requiring surgical procedures had lower rates of CAM use than those who the subjects who did not undergo surgery.

A different set of factors was illustrated in a report of the use of CAM in 86 Scottish children (27). A higher number of corticosteroid courses, higher parental education, and lower patient age were identified as the key factors for the use of CAM in these children. Around two-thirds of the group had used, and 37% were currently using CAM. The most common agents in this setting were probiotics and diary-free diet. Overall, vitamin supplements, herbal remedies, and homeopathy were used less frequently in these children than in the cohorts from North America. Interestingly, however, 89% of the parents in this group reported that they would consider giving CAM in the future.

Probiotics were again reported as the most commonly utilized agent in another North American study involving 236 youngsters with IBD (28). This study recruited children with IBD and children with other chronic disease states. Half of the children with IBD included in this study used CAM, which was greater than rates seen in children with other chronic disease states. Educational achievement, general well-being, European background, and a history of more side-effects from standard drugs were factors linked with CAM use in this group.

One further study specifically ascertained the use of mind-body CAM interventions (yoga, prayer, guided imagery, relaxation, or meditation) in children with IBD (29). Almost two-thirds of the 67 adolescents included in this assessment used prayer for symptom management more than once every week. Relaxation and imagery were more commonly used than meditation or yoga. The girls in this group were more likely to use relaxation techniques regularly, whilst the children with more severe disease would be more likely to consider future use of relaxation than those with less severe activity. There was little relationship between QOL scores (using a generic tool) and use of CAM. Similar to the other pediatric cohorts, the adolescents in this group reported high rates of potential future use of all five types of mind-body CAM.

One important aspect of the use of CAM therapies is clear communication about the use of these agents. It is important for practitioners to be able to introduce the concept of CAM usage within the therapeutic relationship, and also for practitioners to remember to ask about possible usage. Furthermore, if asked about a CAM agent by a patient or parent, then the practitioner should endeavor to answer with an open and non-dismissive approach, in order to maintain clear lines of communication. One New Zealand study ascertained that only 11% of patients could recall having been asked by a doctor if they were using CAM (30). Further, almost one quarter of the patients in our recent questionnaire-based study had not told their doctor about their use of CAM (22). These data highlight the importance of communication in children with IBD who may be using CAM agents.

A recent Canadian report developed a series of specific questions that could be ways to start a conversation about CAM during a consultation (31). One sample question was “What else do you do to support your child’s health?” The authors also outlined a series of specific points to cover during these discussions. The use of these approaches should help to enhance communication about CAM in children with IBD.

Ensuring clear lines of communication about the use of CAM agents is important in regards to safety of these interventions (32). Herbal remedies, for instance, have been associated with hepatic and renal impairment. However, there are not yet clear data on adverse events related to the use of CAM in children with IBD.

## Summary and Conclusion

Overall, CAM agents are commonly administered to children with IBD. The available reports indicate that at least 40% of such children have recently been given CAM, with a smaller number currently using. The usage rates and the types of CAM agents administered differ between the reported studies, with variations within and between countries.

These differences may reflect methodological differences, with different questionnaires or approaches to collection of data. Standardization of questionnaires and the adoption of consistent methods more would ensure more direct comparison within and between regions or countries. Even if such standard approach was adopted, it is likely that variation between countries would persist, reflecting different cultural attitudes to CAM and different patterns of access to various CAM agents.

Furthermore, the available studies highlight differences in the factors reported to influence CAM usage between European and North American cohorts. Again these variations may reflect access to particular CAM agents.

Some data indicates that children with IBD use CAM more frequently than healthy children (2, 4). It is unclear if this is a reflection of the chronic and incurable nature of IBD, or the specific involvement of the GI tract (and consequent bowel symptoms). In addition, increasing rates of CAM usage in children with IBD may reflect changes and attitudes in the wider community.

Probiotics and fish oil are two widely used agents in children with IBD. This may reflect the wider exposure of information about these agents consequent to numerous scientific reports. Even though the data to universally support these therapies is lacking, the fact that these have been subject to scientific scrutiny may have enhanced their profile. Other agents with less awareness or exposure may be utilized less as a consequence.

In conclusion, CAM agents are used frequently by children and adolescents with IBD in almost every reported study. It is likely that rates will continue to increase. Pediatric gastroenterologists caring for children with IBD must ensure adequate personal understanding and awareness of CAM agents, and have a working knowledge of potential roles, possible interactions, and contraindications. Furthermore, pediatric gastroenterologists must remember to ask patients and parents about their use of CAM.

## Conflict of Interest Statement

The authors declare that the research was conducted in the absence of any commercial or financial relationships that could be construed as a potential conflict of interest.
